# Determination of Flavonolignan Compositional Ratios in *Silybum marianum* (Milk Thistle) Extracts Using High-Performance Liquid Chromatography

**DOI:** 10.3390/molecules29132949

**Published:** 2024-06-21

**Authors:** Wei Chen, Xi Zhao, Zhenghua Huang, Shihui Luo, Xuguang Zhang, Wei Sun, Tao Lan, Ruikun He

**Affiliations:** 1 BYHEALTH Institute of Nutrition and Health, Guangzhou 510663, China; chenw8@by-health.com (W.C.);; 2 Shanghai Institute of Nutrition and Health, Chinese Academy of Sciences, Shanghai 200031, China; 3 China National Institute of Standardization, Beijing 100191, China

**Keywords:** compositional ratios, flavonolignan, *Silybum marianum*, milk thistle, HPLC

## Abstract

Milk thistle is one of the most popular ingredients in the liver protection products market. Silymarin is the main component of milk thistle and contains multiple isomers. There have been few studies focusing on the compositional ratios of silymarin isomers. In this study, we developed an HPLC method for the separation and quantification of silymarin isomers, thereby elucidating their compositional ratios. Through the analysis of more than 40 milk thistle extract products on the market, we found that the ratios, specifically Ratio 1 (the silybin B content to the silybin A content, SBNB/SBNA) and Ratio 2 (the sum of the contents of silybin B and isosilybin B to the sum of the contents of silybin A and isosilybin A, (SBNB + IBNB)/(SBNA + IBNA)), are highly consistent across milk thistle extracts, averaging approximately 1.58 and 1.28, respectively. Furthermore, such ratios were verified in milk thistle seed samples. This study introduces significant findings concerning the stable ratios among silymarin isomers in milk thistle extracts and seeds, thereby offering an innovative approach for quality assurance of milk thistle extracts.

## 1. Introduction

*Silybum marianum* (L.) Gaertner, commonly known as milk thistle, is a spiny annual or biennial plant, native to the Mediterranean but now cultivated and growing worldwide [1]. Milk thistle has been cultivated in China since the 1950s, planted in the northeast area for medicinal purpose since the 1970s, and was listed in Chinese Pharmacopoeia in 2005 [2]. Milk thistle is a commercially important herbal plant and is increasingly recognized as a popular ingredient in the herbal supplement industry. Standardized milk thistle extract, derived from the desiccated fruits of *Silybum marianum*, is commercially available in Italy and is recognized for its antihepatotoxic properties [3]. Silymarin, a principal bioactive constituent of milk thistle extract, is recognized for its supportive role in the therapeutic management of hepatic disorders, including acute viral hepatitis, alcoholic liver disease, and chemically-induced liver toxicity [4]. 

**Figure 1:**
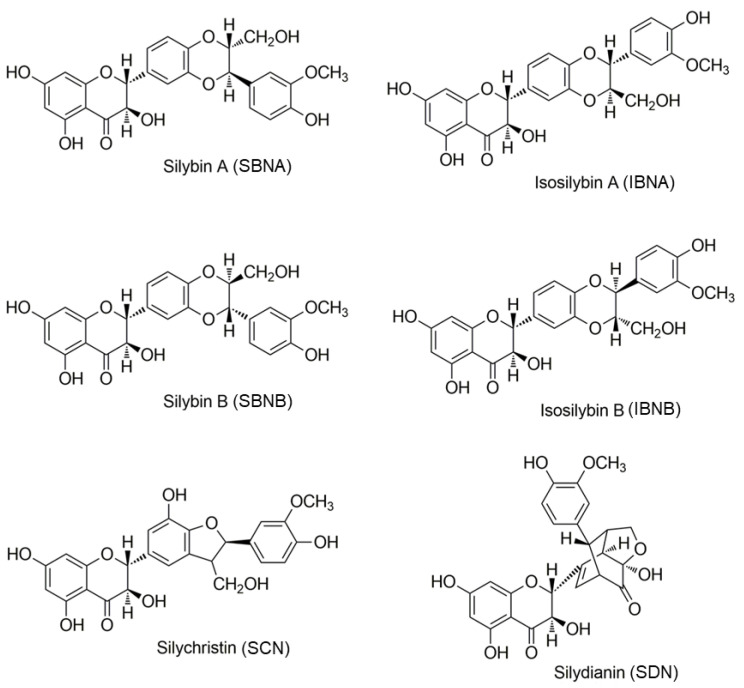
Chemical structure of silymarin flavonolignan isomers (molecular formula: C_25_H_22_O_10_).

Further phytochemical studies on silymarin have suggested that silymarin consists of some flavonolignan isomers (Figure 1), including silybin A (SBNA), silybin B (SBNB), isosilybin A (IBNA), isosilybin B (IBNB), silydianin (SDN), and silychristin (SCN) [5,6]. A standardized milk thistle dry extract is refined and quantified using the nominal silymarin percentage (in the range of 30.0–65.0%) which corresponds to the sum of SBNA, SBNB, IBNA, IBNB, SDN, and SCN concentrations according to the European Pharmacopoeia (EP 10.6) [7]. Among these flavonolignan compounds, silybin is the major component and has the most important biological effects, including hepatoprotective, anti-inflammatory, anti-oxidative, and antifibrotic activities [8]; it accounts for up to 70% of total silymarin content [9]. Most silymarin manufacturers characterize their products by the percentage amounts of silymarin or individual flavonolignan compounds.

Previous published phytochemical studies focused on how to isolate, analyze, or improve the silymarin content by extraction method. There are few reports on the constituent ratios of silymarin compounds in the extracts, although the ratios (if present) of individual compounds are highly important for the quality control of natural milk thistle extract. In this study, we found two stable ratios of flavonolignans in milk thistle extracts which may be used for quality control in the future.

## 2. Results

### 2.1. Valuation of Quantitative Analytical Method

**Figure 2:**
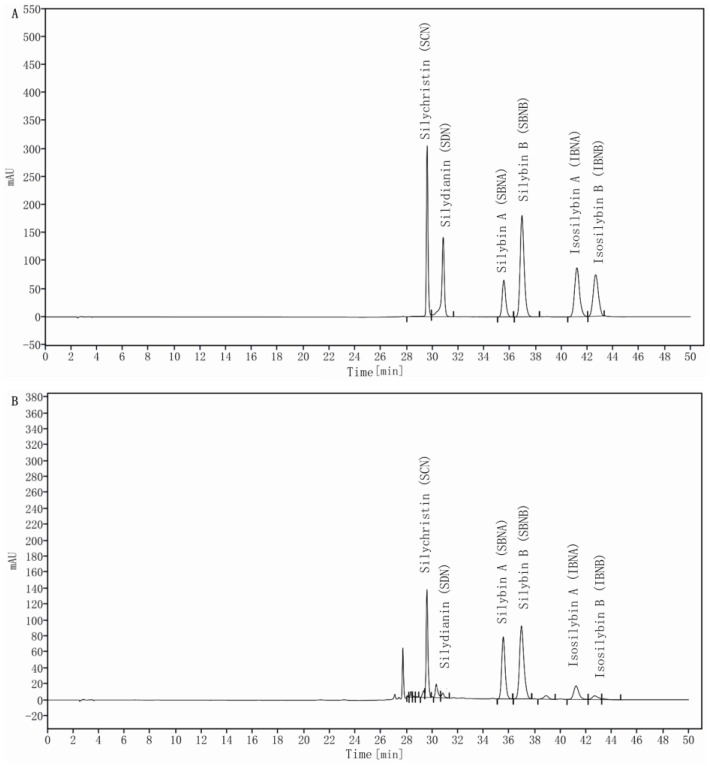
HPLC chromatogram of reference standards (**A**) and milk thistle extract (**B**).

Six silymarin flavonolignans were well separated and retention times (Rt) of SCN, SDN, SBNA, SBNB, IBNA, and IBNB were 29.60, 30.87, 35.65, 37.09, 41.41, and 42.90 min, respectively (Figure 2A). The calibration curves were developed by plotting the integrated peak areas (Y) to the silymarin reference concentrations (X, μg/mL) in a standard solution, using a linear regression approach. Six silymarin standards with five different concentrations were used for this purpose. The regression equations of calibration curves, along with their correlation coefficient, are detailed in Table 1. All correlation coefficients are more than 0.99912, demonstrating excellent linearity. With current chromatographic equipment, the limit of detection (LOD) and limit of quantification (LOQ) for silymarin were established to be within the range of 0.05 to 0.08 μg/mL and 0.13 to 0.21 μg/mL, respectively. These limits were determined based on signal-to-noise ratios of approximately 3 for LOD and 10 for LOQ.

The precision of the method was evaluated by conducting six repeated injections of the prepared mixed standard solutions. The percentages of relative standard deviation (RSD) of peak areas of six reference standards were from 0.36% to 1.11% (Table 2), which showed that the method is precise. Repeatability was performed by injecting six prepared sample solutions. The concentrations of SCN, SDN, SBNA, SBNB, IBNA, and IBNB were calculated, and RSD% of concentrations of six reference standards were from 1.02% to 2.67% (Table 2). The sample solutions were tested on 0th, 12th, 24th, 36th and 48th hour of experiment and solutions were stored at normal storage temperature throughout. RSD% of concentrations of SCN, SDN, SBNA, SBNB, IBNA, and IBNB were 1.07%, 1.27%, 3.20%, 2.72%, 0.70%, and 3.05%, respectively (Table 2). Hence, it can be concluded that the solution is stable for up to 48 h at room temperature.

A recovery experiment was carried out to evaluate the accuracy of the method. The formula for calculating the recovery rate is as follows: recovery (%) = (found amount − original amount)/added amount × 100 [10]. A predetermined quantity (0.2 g) of the sample was spiked with six known amounts of silymarin flavonolignans. The spiked sample was extracted and analyzed according to Section 4.3 and Section 4.4. This process was replicated for two additional samples. The recovery rates of six silymarin flavonolignans are presented in Table 3. The data suggest that the method used in this study is highly accurate, with recovery rates for the flavonolignans ranging from 98.52% to 104.41% and the RSD between 0.05% and 1.23%.

### 2.2. The Contents and Ratios of Individual Compounds in Extract

To evaluate the content of silymarin flavonolignans in milk thistle extracts, accurate quantification was used to analyze the different sources of milk thistle extract samples. Specifically, six silymarin flavonolignans were accurately quantified by their own linear regression equations of standard curves. The total silymarin content is the sum of all the quantified silymarin flavonolignans; Ratio 1 is the ratio of the SBNB content to the SBNA content (Ratio 1 = SBNB/SBNA); Ratio 2 is the ratio of the sum of the contents of SBNB and IBNB to the sum of the contents of SBNA and IBNA (Ratio 2 = (SBNB + IBNB)/(SBNA + IBNA)). 

**Figure 3:**
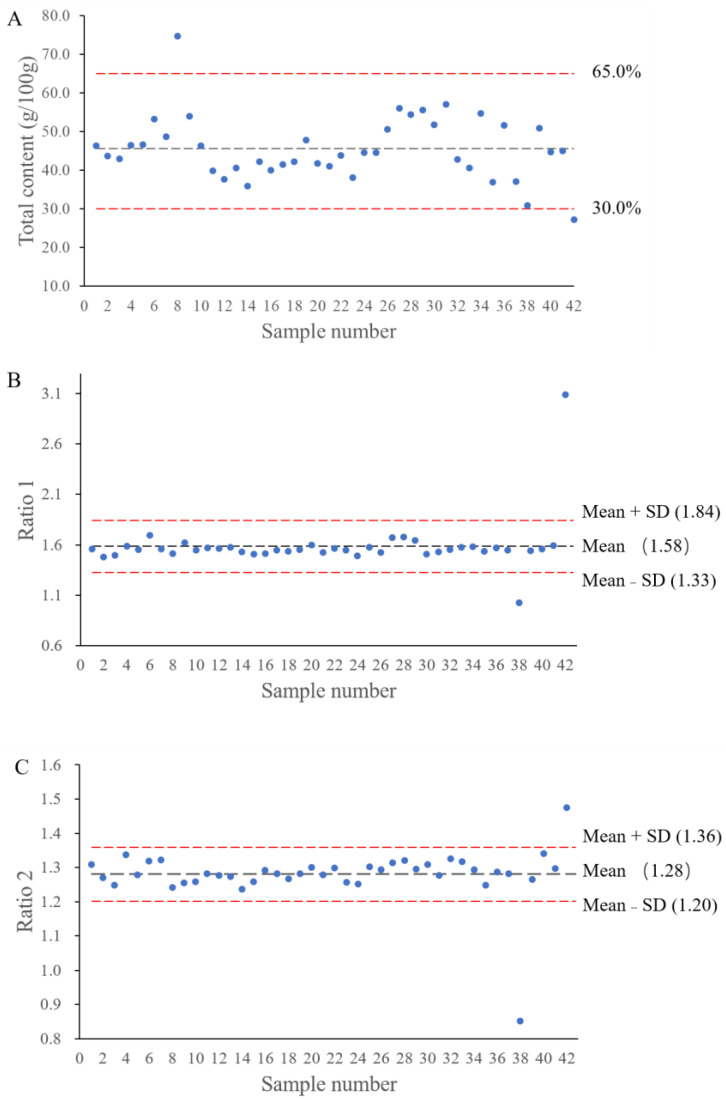
The ranges of total silymarin flavonolignans content (**A**), Ratio 1 (**B**) and Ratio 2 (**C**) in the 42 tested milk thistle extracts.

The concentrations of specified silymarin flavonolignans and their respective ratios are delineated in Table 4. Among the 42 batches of samples, SBNB and SBNA are the major components in the vast majority of commercially available extracts. Both the content of individual compounds and the total silymarin content vary among different sources of milk thistle extracts. Specifically, the contents of SBNB range from 14.02 ± 0.99 to 21.96 ± 1.34 g/100 g in the ethanol extracts, 8.69 ± 0.78 to 22.38 ± 1.49 g/100 g in the ethyl acetate extracts, and 13.78 ± 1.21 to 21.39 ± 1.68 g/100 g in the acetone extracts. The milk thistle extracts contain about 8.42 ± 0.68 to 14.61 ± 1.26 g/100 g SBNA. The total silymarin content ranges from 30.94 ± 2.11 to 57.13 ± 3.61 g/100 g, except for two samples (S8 and S42) that are outside the standard range (30.0–65.0%) of the European Pharmacopoeia (Figure 3A). 

It was found that the ratios of silymarin flavonolignans are relatively stable after analyzing 42 batches of silymarin extracts from different sources (Table 4 and Figure 3). Specifically, the mean of Ratio 1 (SBNB/SBNA) is 1.58, and the range of Ratio 1 is between 1.48 ± 0.01 and 1.70 ± 0.02 after excluding two discrete values (1.01 and 3.09 from samples S38 and S42 respectively). Similarly, the mean of Ratio 2 ((SBNB + IBNB)/(SBNA + IBNA)) is very stable at around 1.28, and it ranges from 1.24 ± 0.01 to 1.34 ± 0.01 after excluding two discrete values (0.85 and 1.48 from samples S38 and S42, respectively). It is worth noting that the ratios calculated using peak areas of silymarin flavonolignans are also very stable, which are slightly lower than those calculated using the contents of silymarin flavonolignans. The mean values of Ratios 1 and 2 calculated using peak areas are 1.54 and 1.25, respectively. 

### 2.3. The Ratios of Individual Compounds in Milk Thistle Seeds

In order to explore whether the stable Ratios 1 and 2 exist in plant materials, we collected milk thistle seeds from different regions and detected the content and ratio of silymarin flavonolignans in silymarin seed materials. As shown in Table 5, the silymarin contents in samples TC08 and TC09 are different, indicating that milk thistle seeds from different sources contain different amounts of flavonolignans. In sample TC08, the silymarin content obtained by ethanol extraction (1.32 ± 0.02 g/100 g) is similar to that obtained by acetone extraction (1.43 ± 0.02 g/100 g), while the silymarin content obtained by ethyl acetate extraction is much lower (0.57 ± 0.01 g/100 g). Similar results are observed in sample TC09, suggesting that the extraction efficiency of silymarin is affected by different extraction solvents. Surprisingly, although the content of silymarin can be affected by the extraction processes and origin of milk thistle materials, the ratios of silymarin flavonolignans are still stable, almost the same as the ratios of silymarin compounds in the commercial silymarin extract. Specifically, Ratios 1 and 2 of milk thistle seeds are from 1.59 to 1.87, and 1.24 to 1.31, respectively. 

## 3. Discussion

In this study, we found stable ratios among the silymarin content within milk thistle extracts. Additionally, we observed that these ratios are also reflective of those present within the plant material itself, especially Ratio 2, which demonstrated consistency across both the extracts and the plant materials. The occurrence of specific ratio relationships among plant compounds is a recognized phenomenon, with isomeric compounds frequently exhibiting such stable ratios in nature. It is known that tetrahydropalmatine (THP) has two enantiomers (L-THP and D-THP), naturally coexisting in *Rhizoma Corydalis* (Yan Hu Suo, YHS) as a racemic mixture. Gou et al. found that the ratio of peak area of D-THP and L-THP was stable at 60:40 in chromatograms [11]. They further reported that the peak area ratios of two enantiomers in YHS products would vary remarkably when low-quality or crudely extracted YSH materials are used in the production process, or when synthetic raw chemicals are added to meet the standards. To tackle this issue, the authors established a novel strategy for assessing and ensuring the quality of Chinese patent medicine according to the analysis of the ratio of chiral isomers [11].

A study established an HPLC-UV and LC-MS-MS method and quantified the concentration of silymarin components extracted from seeds and marketed milk thistle products (capsules and tincture), including two seed samples and three marketed products [12]. After calculating the ratios based on those published data, we found that the ratios of SBNB/SBNA (Ratio 1 in this study) in seed samples and marketed product samples range from 1.66 to 1.78, and from 1.51 to 1.74, respectively, which is highly consistent with our results. Furthermore, another study analyzed six commercial silymarin products from the USA, China, Italy, and Germany. Although the contents of flavonolignan compounds were different and the total silymarin amounts ranged from 68.3 mg/g to 777.1 mg/g [13], the Ratios 1 (SBNB/SBNA) and 2 ((SBNB + IBNB)/(SBNA + IBNA)), which are obtained based on calculating ratios of individual silymarin contents from literature data, were between 1.42 and 1.70, and 1.09 and 1.28, respectively, which is relatively stable and similar to our findings in this study. 

Langen et al. used gas chromatography with tandem mass spectrometric detection to analyze α-ionone, β-ionone, and β-damascenone in wine and found that the ratio of (R)-enantiomer to (S)-enantiomer of α-ionone was relatively stable and between 45:55 and 52:48 [14]. The author concluded that an adulteration could be assumed if R/S ratios are considerably below 70:30 [14]. Perhaps Ratios 1 and 2 found in this study can be used to identify naturally extracted milk thistle extracts, to distinguish from cases of artificial adulteration.

## 4. Materials and Methods

### 4.1. Milk Thistle Samples 

Sample collection: A total of 42 milk thistle extract samples were obtained from a diverse array of manufacturers, including six local Chinese and two European facilities. These extracts were manufactured using different solvents, specifically, 19 batches were extracted by ethanol, 13 batches were extracted by ethyl acetate, and 10 batches were extracted by acetone. 

Dried milk thistle seed samples: Three samples of milk thistle seeds from Yunnan (batch no. TCYF2023109), Hebei (batch no. TCYF2023108) and Jiangsu (batch no. TCYF2023107) provinces were purchased from the traditional Chinese medicine market.

### 4.2. Chemicals

The reference standards including SBNA (batch no. G23030090), SBNB (batch no. G23030090), IBNA (batch no. G23030071), IBNB (batch no. G23030070), SDN (batch no. G23030087), and SCN (batch no. G23030086) were purchased from TM standard (Beijing, China), and the purities of reference standards were no less than 99.0%. HPLC-grade methanol (MeOH) was purchased from Merck (Phillipsburg, NJ, USA). Water was obtained from a Milli-Q Ultra-pure water system (Millipore, Billerica, MA, USA). Other reagents used in this study were of analytical grade.

### 4.3. Preparation of Samples and Reference Standards

Extract sample preparation: An approximately 0.2 g sample of each milk thistle extract was accurately weighed and reconstituted with 20 mL methanol, followed by vortex mixing for 5 min, ultrasonication for 30 min, and centrifugation at 10,000 rpm for 15 min. The supernatant was diluted 20-fold with methanol and passed through a 0.45 μm filter membrane before HPLC analysis. 

Milk thistle seeds extraction: The dried milk thistle seeds were pulverized and a sample of about 1.0 g was reflux extracted with 100 mL ethanol for 1 h. The filtered residue underwent a second extraction, and the two extraction liquids were combined and passed through a 0.45 μm filter membrane before HPLC analysis. Milk thistle seeds were extracted with MeOH, acetone, and ethyl acetate using similar extraction processes.

Reference standards preparation: A set of six reference standards, including SBNA at a concentration of 0.6694 mg/mL, SBNB at 1.0039 mg/mL, IBNA at 0.9021 mg/mL, IBNB at 0.8699 mg/mL, SDN at 1.0407 mg/mL, and SCN at 1.0279 mg/mL, were each dissolved in MeOH. These individual solutions were subsequently combined and further diluted with MeOH to obtain a range of mixed standard solutions with different concentrations. Prior to HPLC analysis, the solutions underwent filtration through a 0.45 μm membrane filter.

### 4.4. HPLC Conditions

The analysis was conducted using an Agilent 1260 liquid chromatograph (Agilent, MA, USA), which is outfitted with a quaternary pump, an online degasser, an automated sampler, and a diode-array detector. The flavonolignans extracted from milk thistle were separated by an Eclipse XDB C18 column (4.6 mm × 250 mm, 5 μm) provided by Agilent, USA, and maintained at a column temperature of 30 °C. The chromatographic separation was achieved using a binary gradient elution system with solvent A being 1% acetic acid in water and solvent B being 1% acetic acid in MeOH. The gradient elution program was designed with the following timeline: from 0 to 20 min, a linear increase from 5% to 20% B; from 20 to 25 min, a linear increase from 20% to 50% B; from 25 to 40 min, a constant 50% B; a sharp decrease from 50% to 5% B between 40 and 40.1 min; and a return to 5% B from 40.1 to 50 min. The flow rate was maintained at 1.0 mL/min, the detection wavelength was set at 288 nm, and the sample injection volume was set to 10 μL.

## 5. Conclusions

The present study provides an analytical approach for quantification and assessment of silymarin isomers within milk thistle extracts. After analyzing more than 40 batches of samples, we found that even the content of flavonolignans can be affected by the extraction processes and origin of milk thistle materials, and the ratios of flavonolignans are highly consistent. Specifically, Ratio 1 (SBNB/SBNA) and Ratio 2 ((SBNB + IBNB)/(SBNA + IBNA)) are respectively around 1.58 and 1.28 in milk thistle extracts. The identified stable flavonolignan ratios provide a robust metric for quality control within the industry, ensuring the therapeutic integrity and standardization of milk thistle products.

## Figures and Tables

**Table 1:** The regression equations, linear ranges, LOD and LOQ of six silymarin flavonolignans.

Compounds	Calibration Curve	R^2^	Concentration Range (μg/mL)	LOD (μg/mL)	LOQ (μg/mL)
SCN	Y = 25.827X − 1.953	0.99985	2.570–154.185	0.05	0.13
SDN	Y = 20.024X − 6.456	0.99997	2.602–156.105	0.08	0.21
SBNA	Y = 29.147X − 18.342	0.99979	2.425–72.750	0.08	0.20
SBNB	Y = 28.431X − 20.959	0.99912	7.575–227.250	0.08	0.20
IBNA	Y = 25.826X + 1.985	0.99999	2.255–135.315	0.07	0.18
IBNB	Y = 25.827X − 1.953	0.99985	2.570–154.185	0.05	0.13

Y is the integrated peak area and X is the amount of analyte. LOD, limit of detection; LOQ, limit of quantification.

**Table 2:** Precision, repeatability, stability of the HPLC method.

Compounds	RSD (%)		
	Precision	Repeatability	Stability
SCN	0.36	1.22	1.07
SDN	1.09	2.33	1.27
SBNA	1.04	2.76	3.20
SBNB	0.31	1.02	2.72
IBNA	0.24	2.67	0.70
IBNB	1.11	2.05	3.05

**Table 3:** The recovery of six silymarin flavonolignans.

Compounds	Original (mg)	Added (mg)	Found (mg)	Recovery (%)	RSD (%)
SCN	20.08	8.79	29.22	101.21	0.52
	20.62	8.75	29.54	100.58	
	20.60	8.77	29.42	100.17	
SDN	1.91	2.13	4.19	103.71	0.40
	1.96	2.12	4.23	103.68	
	1.96	2.12	4.26	104.41	
SBNA	56.30	11.52	68.24	100.62	0.66
	58.73	11.54	69.92	99.50	
	56.63	11.30	68.38	100.66	
SBNB	57.83	22.41	80.48	100.30	0.37
	59.40	22.32	82.16	100.54	
	59.33	22.35	81.52	99.80	
IBNA	7.28	3.27	10.58	100.28	0.05
	7.48	3.26	10.77	100.28	
	7.47	3.27	10.78	100.37	
IBNB	2.73	1.25	3.93	98.74	1.23
	2.80	1.25	4.08	100.74	
	2.80	1.25	3.99	98.52	

**Table 4:** The contents and ratios of silymarin flavonolignans of milk thistle extracts (mean ± SD).

Samples	SCN (g/100 g)	SDN (g/100 g)	SBNA (g/100 g)	SBNB (g/100 g)	IBNA (g/100 g)	IBNB (g/100 g)	Total (g/100 g)	Ratio 1	Ratio 2
Ethanol extracts of milk thistle									
S1	9.32 ± 0.78	1.02 ± 0.03	12.29 ± 0.99	19.15 ± 1.23	3.28 ± 0.10	1.23 ± 0.04	46.29 ± 3.55	1.56 ± 0.01	1.31 ± 0.01
S2	8.50 ± 0.66	0.82 ± 0.03	12.19 ± 0.78	18.03 ± 1.11	2.97 ± 0.09	1.23 ± 0.05	43.73 ± 3.43	1.48 ± 0.01	1.27 ± 0.01
S4	8.40 ± 0.54	1.13 ± 0.05	12.39 ± 0.89	19.66 ± 1.24	3.38 ± 0.12	1.43 ± 0.02	46.50 ± 3.46	1.59 ± 0.01	1.34 ± 0.01
S6	13.36 ± 1.12	0.81 ± 0.03	12.35 ± 0.76	20.95 ± 1.32	4.45 ± 0.21	1.21 ± 0.03	53.24 ± 3.99	1.70 ± 0.02	1.32 ± 0.01
S7	9.53 ± 0.76	1.87 ± 0.08	12.64 ± 0.75	19.69 ± 1.22	3.42 ± 0.29	1.55 ± 0.04	48.70 ± 3.45	1.56 ± 0.01	1.32 ± 0.01
S8	15.77 ± 1.32	3.69 ± 0.12	18.74 ± 1.22	28.37 ± 2.01	5.94 ± 0.34	2.25 ± 0.12	74.66 ± 5.41	1.51 ± 0.01	1.24 ± 0.01
S12	9.28 ± 0.65	1.13 ± 0.04	8.97 ± 0.67	14.02 ± 0.99	2.99 ± 0.21	1.24 ± 0.05	37.74 ± 2.89	1.56 ± 0.01	1.28 ± 0.01
S15	9.36 ± 0.61	1.34 ± 0.04	10.80 ± 0.77	16.26 ± 1.00	3.19 ± 0.22	1.34 ± 0.06	42.19 ± 3.32	1.50 ± 0.01	1.26 ± 0.01
S20	9.44 ± 0.54	1.45 ± 0.04	10.07 ± 0.75	16.09 ± 0.98	3.42 ± 0.19	1.45 ± 0.06	41.82 ± 3.12	1.60 ± 0.01	1.30 ± 0.01
S22	9.00 ± 0.23	1.12 ± 0.07	11.56 ± 0.81	18.10 ± 1.02	3.17 ± 0.19	1.02 ± 0.01	43.87 ± 3.21	1.57 ± 0.01	1.30 ± 0.01
S23	8.29 ± 0.11	1.31 ± 0.05	9.61 ± 0.56	14.87 ± 0.77	3.03 ± 0.21	1.01 ± 0.02	38.13 ± 3.01	1.55 ± 0.01	1.26 ± 0.01
S24	9.09 ± 0.34	2.12 ± 0.09	11.52 ± 0.72	17.17 ± 1.02	3.33 ± 0.24	1.41 ± 0.03	44.55 ± 3.24	1.49 ± 0.01	1.25 ± 0.01
S27	13.56 ± 0.98	1.52 ± 0.08	13.16 ± 0.67	21.96 ± 1.34	4.55 ± 0.32	1.32 ± 0.03	56.06 ± 3.56	1.67 ± 0.02	1.31 ± 0.01
S28	13.17 ± 0.89	1.52 ± 0.09	12.77 ± 0.79	21.38 ± 1.27	4.36 ± 0.29	1.22 ± 003	54.42 ± 3.49	1.67 ± 0.02	1.32 ± 0.01
S29	12.98 ± 0.78	1.62 ± 0.08	13.28 ± 0.82	21.80 ± 1.31	4.56 ± 0.31	1.32 ± 0.04	55.56 ± 3.43	1.64 ± 0.01	1.30 ± 0.01
S30	10.12 ± 0.67	1.02 ± 0.04	14.32 ± 0.91	21.58 ± 1.34	3.27 ± 0.28	1.43 ± 0.04	51.75 ± 3.37	1.51 ± 0.00	1.31 ± 0.01
S32	9.55 ± 0.54	1.75 ± 0.05	10.79 ± 0.66	16.74 ± 0.76	2.77 ± 0.19	1.23 ± 0.02	42.84 ± 3.27	1.55 ± 0.01	1.33 ± 0.01
S37	8.17 ± 0.43	1.34 ± 0.06	9.31 ± 0.65	14.38 ± 0.66	2.79 ± 0.22	1.14 ± 0.02	37.03 ± 2.64	1.54 ± 0.01	1.28 ± 0.01
S40	8.24 ± 0.68	1.03 ± 0.05	12.36 ± 0.89	19.27 ± 1.05	2.78 ± 0.26	1.03 ± 0.02	44.71 ± 3.25	1.56 ± 0.01	1.34 ± 0.01
Ethyl acetate extracts of milk thistle									
S9	10.86 ± 0.59	6.86 ± 0.58	11.57 ± 0.94	18.74 ± 1.25	4.51 ± 0.32	1.43 ± 0.02	53.97 ± 3.21	1.62 ± 0.02	1.25 ± 0.01
S10	10.22 ± 0.61	1.75 ± 0.09	11.66 ± 0.87	18.06 ± 1.32	3.51 ± 0.21	1.03 ± 0.01	46.34 ± 3.01	1.55 ± 0.01	1.26 ± 0.01
S11	8.98 ± 0.55	2.27 ± 0.21	9.60 ± 0.66	15.07 ± 1.11	2.89 ± 0.18	0.93 ± 0.00	39.85 ± 3.05	1.57 ± 0.01	1.28 ± 0.01
S14	8.94 ± 0.59	1.25 ± 0.04	8.42 ± 0.68	12.89 ± 0.94	3.01 ± 0.19	1.25 ± 0.01	35.85 ± 2.78	1.53 ± 0.01	1.24 ± 0.01
S19	9.29 ± 0.63	2.48 ± 0.17	12.28 ± 1.02	19.09 ± 1.54	3.51 ± 0.21	1.14 ± 0.01	47.88 ± 3.59	1.55 ± 0.01	1.28 ± 0.01
S21	8.92 ± 0.54	1.03 ± 0.04	10.76 ± 0.93	16.40 ± 1.27	2.87 ± 0.20	1.03 ± 0.01	41.10 ± 2.99	1.52 ± 0.01	1.28 ± 0.01
S25	8.93 ± 0.52	0.91 ± 0.01	11.87 ± 0.88	18.67 ± 1.33	3.25 ± 0.19	1.01 ± 0.01	44.64 ± 3.11	1.57 ± 0.01	1.30 ± 0.01
S26	9.43 ± 0.49	0.81 ± 0.02	14.29 ± 1.23	21.79 ± 1.56	3.34 ± 0.21	1.01 ± 0.01	50.68 ± 3.68	1.52 ± 0.01	1.29 ± 0.01
S31	11.45± 0. 69	2.96 ± 0.11	14.61 ± 1.26	22.38 ± 1.49	4.19 ± 0.26	1.64 ± 0.02	57.13 ± 3.61	1.53 ± 0.01	1.28 ± 0.01
S33	9.55 ± 0.73	1.85 ± 0.09	9.85 ± 0.88	15.50 ± 0.98	2.77 ± 0.17	1.13 ± 0.01	40.65 ± 2.78	1.57 ± 0.01	1.32 ± 0.01
S36	11.28 ± 0.81	2.05 ± 0.09	13.03 ± 1.02	20.41 ± 1.51	3.79 ± 0.21	1.23 ± 0.02	51.69 ± 3.51	1.57 ± 0.01	1.29 ± 0.01
S38	8.59 ± 0.69	1.66 ± 0.06	8.49 ± 0.99	8.69 ± 0.78	2.69 ± 0.18	0.83 ± 0.01	30.94 ± 2.11	1.02 ± 0.00	0.85 ± 0.00
S41	9.21 ± 0.51	1.74 ± 0.04	11.35 ± 0.87	18.11 ± 1.26	3.48 ± 0.27	1.13 ± 0.01	45.11 ± 3.48	1.59 ± 0.02	1.30 ± 0.01
Acetone extracts of milk thistle									
S3	8.40 ± 0.54	1.13 ± 0.01	11.78 ± 0.87	17.62 ± 1.25	3.07 ± 0.21	0.92 ± 0.01	42.91 ± 3.21	1.50 ± 0.01	1.25 ± 0.01
S5	10.45 ± 0.41	1.84 ± 0.03	11.47 ± 0.98	17.82 ± 1.41	3.58 ± 0.20	1.43 ± 0.01	46.70 ± 3.33	1.55 ± 0.01	1.28 ± 0.01
S13	9.38 ± 0.49	1.34 ± 0.02	9.99 ± 0.76	15.77 ± 1.11	3.19 ± 0.19	1.03 ± 0.01	40.70 ± 3.01	1.58 ± 0.01	1.27 ± 0.01
S16	8.81 ± 0.51	2.69 ± 0.05	9.12 ± 0.71	13.78 ± 1.21	3.32 ± 0.26	2.28 ± 0.09	40.10 ± 3.21	1.51 ± 0.01	1.29 ± 0.01
S17	9.04 ± 0.59	1.75 ± 0.04	10.48 ± 0.91	16.23 ± 1.39	2.98 ± 0.21	1.03 ± 0.01	41.49 ± 3.36	1.55 ± 0.01	1.28 ± 0.01
S18	9.32 ± 0.43	2.56 ± 0.05	10.34 ± 0.84	15.87 ± 1.51	3.07 ± 0.21	1.13 ± 0.02	42.28 ± 3.14	1.53 ± 0.01	1.27 ± 0.01
S34	11.90 ± 0.61	1.92 ± 0.06	13.52 ± 1.11	21.39 ± 1.68	4.34 ± 0.31	1.71 ± 0.02	54.68 ± 3.54	1.58 ± 0.01	1.29 ± 0.01
S35	8.77 ± 0.48	1.53 ± 0.03	9.17 ± 0.83	14.07 ± 1.21	2.75 ± 0.27	0.82 ± 0.01	37.00 ± 3.12	1.53 ± 0.01	1.25 ± 0.01
S39	11.38 ± 0.67	1.03 ± 0.01	13.22 ± 0.91	20.40 ± 1.81	3.79 ± 0.35	1.13 ± 0.02	50.85 ± 3.75	1.54 ± 0.01	1.27 ± 0.01
S42	10.82 ± 0.58	1.21 ± 0.02	2.33 ± 0.03	7.18 ± 0.41	3.84 ± 0.37	1.92 ± 0.03	27.31 ± 2.21	3.09 ± 0.07	1.48 ± 0.02

**Table 5:** The contents and ratios of silymarin flavonolignans of milk thistle seeds (mean ± SD).

Samples	Extraction Solvent	SCN (g/100 g)	SDN (g/100 g)	SBNA (g/100 g)	SBNB (g/100 g)	IBNA (g/100 g)	IBNB (g/100 g)	Total (g/100 g)	Ratio 1	Ratio 2
TC08	EtOH	0.20 ± 0.00	0.45 ± 0.01	0.15 ± 0.00	0.28 ± 0.00	0.14 ± 0.00	0.10 ± 0.00	1.32 ± 0.02	1.87 ± 0.01	1.31 ± 0.01
	acetone	0.19 ± 0.00	0.52 ± 0.01	0.17 ± 0.00	0.30 ± 0.00	0.15 ± 0.00	0.10 ± 0.00	1.43 ± 0.02	1.76 ± 0.01	1.25 ± 0.01
	EtOAc	0.08 ± 0.00	0.19 ± 0.00	0.07 ± 0.00	0.13 ± 0.00	0.06 ± 0.00	0.04 ± 0.00	0.57 ± 0.01	1.86 ± 0.01	1.31 ± 0.01
TC09	EtOH	0.45 ± 0.01	0.35 ± 0.01	0.39 ± 0.01	0.66 ± 0.01	0.19 ± 0.00	0.10 ± 0.00	2.14 ± 0.03	1.69 ± 0.01	1.31 ± 0.01
	acetone	0.49 ± 0.01	0.42 ± 0.01	0.46 ± 0.01	0.78 ± 0.02	0.23 ± 0.00	0.11 ± 0.00	2.49 ± 0.03	1.70 ± 0.01	1.29 ± 0.01
	EtOAc	0.28 ± 0.00	0.26 ± 0.01	0.29 ± 0.00	0.49 ± 0.01	0.14 ± 0.00	0.06 ± 0.00	1.52 ± 0.02	1.69 ± 0.01	1.28 ± 0.01
TC10	MeOH	0.71 ± 0.02	0.10 ± 0.00	0.68 ± 0.02	1.08 ± 0.04	0.26 ± 0.01	0.09 ± 0.00	2.92 ± 0.03	1.59 ± 0.01	1.24 ± 0.01

EtOH: ethanol; EtOAc: ethyl acetate; MeOH: methanol.

## Data Availability

The data presented in this study are available on request from the corresponding author.
